# Integrating Macrophages into Organotypic Co-Cultures: A 3D In Vitro Model to Study Tumor-Associated Macrophages

**DOI:** 10.1371/journal.pone.0040058

**Published:** 2012-07-06

**Authors:** Nina Linde, Claudia M. Gutschalk, Claudia Hoffmann, Dilan Yilmaz, Margareta M. Mueller

**Affiliations:** 1 Group Tumor and Microenvironment, German Cancer Research Center (DKFZ), Heidelberg, Germany; 2 Hochschule Furtwangen University, Campus Villingen-Schwenningen, Germany; Thomas Jefferson University, United States of America

## Abstract

Tumor progression is controlled by signals from cellular and extra-cellular microenvironment including stromal cells and the extracellular matrix. Consequently, three-dimensional in vitro tumor models are essential to study the interaction of tumor cells with their microenvironment appropriately in a biologically relevant manner. We have previously used organotypic co-cultures to analyze the malignant growth of human squamous cell carcinoma (SCC) cell lines on a stromal equivalent in vitro. In this model, SCC cell lines are grown on a collagen-I gel containing fibroblasts. Since macrophages play a critical role in the progression of many tumor types, we now have expanded this model by integrating macrophages into the collagen gel of these organotypic tumor co-cultures. This model was established as a murine and a human system of skin SCCs. The effect of macrophages on tumor progression depends on their polarization. We demonstrate that macrophage polarization in organotypic co-cultures can be modulated towards and M1 or an M2 phenotype by adding recombinant IFN-γ and LPS or IL-4 respectively to the growth medium. IL-4 stimulation of macrophage-containing cultures resulted in enhanced tumor cell invasion evidenced by degradation of the basement membrane, enhanced collagenolytic activity and increased MMP-2 and MMP-9. Interestingly, extended co-culture with tumor cells for three weeks resulted in spontaneous M2 polarization of macrophages without IL-4 treatment. Thus, we demonstrate that macrophages can be successfully integrated into organotypic co-cultures of murine or human skin SCCs and that this model can be exploited to analyze macrophage activation towards a tumor supporting phenotype.

## Introduction

Tumor progression does not only depend on the state of the tumor cells but is influenced by the tumor microenvironment [1], [2]. This microenvironment consists of stromal cells such as fibroblasts, inflammatory cells as well as a tumor specific extracellular matrix. Additionally, three-dimensional (3D) tumor-tumor and tumor-matrix interactions play an important role in tumor regulation. Appreciating this, it becomes clear that complex interactions govern the development of tumors in vivo. As a consequence, sophisticated in vitro models are required that allow investigation of tumor-stroma interaction in an in vivo like environment. Different approaches have been taken to tackle this issue. Tumor spheroids are used as a model for 3D tumor growth. Integration of other cell types such as endothelial cells allows to integrate tumor-stroma interactions in this model (reviewed by Hirschberger et al. [3]). Tumor spheroids can be performed at a high-throughput scale and are useful tools for screening experiments. Yet, they do not fully acknowledge the tissue architecture of the host tissue that the tumors are derived from. This is better accomplished by tissue sections which are cultivated as ex vivo organotypic cultures (OTCs) [4]. However, tissue section OTCs suffer from inter-individual differences in each isolation and are limited to the organization of the original tissue as it is at the current state since they do not allow the integration of cells of different origin to analyze basic questions of tumor-stroma interactions in establishment and maintenance. In contrast, organotypic co-cultures, in which the different cellular components are put together in a matrix, re-assemble a tissue in vitro and thus maintain the tissue context while they are still easily accessible for experimental manipulation. N.E. Fusenig and co-workers have previously established a three-dimensional organotypic co-culture system as a physiological model for skin [5]–[7]. In this model, keratinocytes are grown at the air-liquid interface on top of a fibroblast containing dermal equivalent - precisely a collagen-I gel - where they form a squamous and differentiated epithelium. Fibroblasts are needed for the formation of a functional skin equivalent by contributing to a reciprocally stimulating feedback loop that allows keratinocyte growth and differentiation [8]. Thus, even though organotypic skin equivalents are simplified culture models, they permit a far-reaching recapitulation of keratinocyte differentiation and epidermal and dermal interaction in vitro. Subsequently, by using a scaffold matrix instead of collagen-I, the OTC model was further developed by Boukamp and co-workers to mimic the steady state tissue context in normal skin [9]. Additionally, we adapted the collagen-I model that rather mimics a wound-like situation as an in vitro model for carcinoma growth by including malignant cells such as skin SCC cells [10], of the head and neck [11], or Lewis Lung Cancer cells [unpublished data] instead of normal keratinocytes. Despite the increasing independence of tumor cells from their microenvironment, it is well established that fibroblasts critically contribute to malignant tumor growth [12]. In agreement with this, inclusion of fibroblasts into organotypic tumor co-cultures critically contributes to invasive growth [13].

However, formation of a tumor supportive microenvironment in vivo requires not only the interaction between fibroblasts and tumor cells. It is now widely accepted that inflammatory cells and specifically macrophages are important contributors to a tumor supporting stroma. They are recruited by all solid tumors and critically contribute to tumor progression by secretion of proteases, angiogenic substances and growth factors [14], [15]. This effect depends on the activation status of macrophages. Within the tumor microenvironment macrophages undergo polarization resulting in an alternatively activated M2 macrophage population [15], [16], which then exerts the tumor-promoting effects described above. Cytokines inducing this M2 polarization are IL-4, IL-10 and IL-13 [17].

So far, the investigation of tumor-promoting effects of macrophages was restricted to simple co-culture systems or the analysis of in vivo experiments. For this reason we established a protocol where bone-marrow derived or peripheral blood derived macrophages respectively are integrated into the model of 3D organotypic tumor co-cultures. This allows the investigation of their role for tumor promotion of SCCs in vitro in a complex three-dimensional model mimicking the in vivo situation and thus provides a useful simplified tool for mechanistic experiments. We have established this model system as a murine as well as a human system, to allow the detailed functional study of human tumor-associated macrophages in a complex in vivo like environment. Using this model system, we demonstrate that the presence of M2 polarized macrophages that develop either after stimulation with IL-4 or upon extended co-culture with tumor cells and fibroblasts supports invasive growth of the SCC tumor cells in OTCs.

## Materials and Methods

### Cell Lines

PDVA cells were generated by *in vivo* DMBA-treatment of B10LP mouse keratinocytes as described [18] and maintained in DMEM with 10% FCS (Gibco, Invitrogen) and 1% penicillin/streptomycin (PAA). A-5RT3 cells resulted from in vivo passaging of benign A-5 cells as described [19] and were maintained in 4×MEM with 10% FCS (Gibco, Invitrogen), 1% penicillin/streptomycin (PAA) and G418 (PAA). All cells were routinely tested for mycoplasma contamination as described [20] and always found to be negative.

### Preparation of Murine Bone-marrow Derived Macrophages

Bone marrow was isolated from tibia and femur from C57Bl6/N mice. Fibroblasts were removed by plastic adherence overnight. Cells in the supernatant were cultured in presence of L929-cell conditioned medium (LCCM) containing M-CSF. To obtain LCCM, culture supernatant of L929 cells was harvested daily for five subsequent days. The conditioned medium was sterile-filtrated and mixed with the culture medium in a ratio of 1∶5. Purity of the resulting macrophages was confirmed by flow cytometry (>90% CD11b^+^/F4/80^+^). All procedures performed on animals were approved by the local government (approval committee: Regierungspräsidium Karlsruhe, approval ID: AZ #35-9185.81/G-152/08).

### Preparation of Human Macrophages from Peripheral Blood Monocytes

Human peripheral blood monocytes (PBMCs) were isolated from buffy coats (Staedtisches Klinikum Karlsruhe, Germany) by Hypaque-Ficoll density gradient centrifugation. PBMCs were differentiated to macrophages by cultivation in RPMI-1640 with 25 ng/ml M-CSF (R&D) for six days. Purity of macrophages were controlled by flow cytometry (>90% CD68^+^).

### Fibroblast Isolation

Primary human dermal fibroblasts were isolated as described [7]. Primary murine dermal fibroblasts were isolated from back skin samples of adult CD-1 nude mice. Skin samples were washed in PBS, cut into small pieces and placed in a 10 cm tissue culture dishes where they were covered with sterile cover slips. After addition of medium (DMEM, 10% FCS, 1% penicillin/streptomycin), fibroblasts grew out of the explants within 3–4 days. Remaining colonies of epidermal keratinocytes were eliminated by plastic adherence, i.e. fibroblasts were passaged using trypsin but no EDTA leaving keratinocytes behind. No keratinocytes could be detected in the fibroblast cultures after two passages.

All procedures performed on animals were approved by the local government (approval committee: Regierungspräsidium Karlsruhe, approval ID: AZ #35-9185.81/G-152/08).

### Organotypic Co-cultures

For the preparation of dermal equivalents, a native type I rat collagen solution (2 mg/mL collagen-I, 10% Hank’s, 10% FCS) was supplemented with either 2.5* 10^5^ dermal fibroblasts or 2.5* 10^5^ macrophages or a mixture of 1.25*10^5^ macrophages and 1.25*10^5^ fibroblasts. Of this mixture, 2.5 mL was poured into membrane filter inserts (Falcon, Becton Dickinson, Heidelberg, Germany), placed in deep six-well trays (Becton Dickinson) and left to polymerize at 37°C. Further preparation was as described [11]. All co-cultures were maintained in DMEM supplemented with 10% FCS, 1% penicillin/streptomycin, 50 µg/ml ascorbic acid (Sigma, Munich, Germany) and 10 ng/ml M-CSF (10 ng/ml, R&D, Darmstadt, Germany). Medium was exchanged three times a week. IL-4 (25 ng/ml, R&D) was added to the medium to induce M2 polarization of macrophages. For M1 polarization, LPS (100 ng/ml, Invitrogen) and IFN-γ (50 ng/ml, R&D) were added to the medium during the second week. For 3 weeks, two cultures per week were taken out, cut into halves and processed for histology as wells as cryostat sectioning. Data are representative of two independent experiments with two replicas each.

### Histological Analysis

Half of the cultures were fixated in 3.7% formaldehyde/PBS at 4°C. Further preparation and hemalaun/eosin (H&E) stainings were as described [2]. Microscopic analysis was performed with an Olympus BX51 microscope and a color camera (color view, Olympus) applying the Cell software (Olympus).

### Indirect Immunofluorescent Analysis

Cryosections (6 µm) were dried on slides, fixated and stained as described [21]. For combined stainings of two rat monoclonal antibodies (CD-206 and F4/80), fluorophore-conjugated antibodies were used. The following primary antibodies were used: F4/80, rat (Dianova; clone BM8); F4/80-FITC (Biolegend, Uithoorn, The Netherlands, clone BM8); Lyve-1, rabbit (DCS, Hamburg, Germany; polyclonal); CD-206-A647 (Biolegend; clone MR5D3); collagen-IV, rabbit (Progen; polyclonal); laminin, rabbit (Progen; polyclonal); alpha-6 Integrin, rat (kind gift from Dr. Dirk Breitkreutz, monoclonal); MMP-2, MMP-9 (both goat polyclonal, R&D); CD68, mouse (Dianova, clone Ki-M7); CD-209, rabbit (Abcam, Cambridge, UK, polyclonal); CD127, rabbit (Abcam, polyclonal). Nuclei were stained with 10 µg/ml Hoechst 33258/bisbenzimide together with the secondary antibodies. Secondary antibodies used were: donkey-anti-rat-Dyelight-488, donkey-anti-rat-Cy3, donkey-anti-rabbit-Dyelight-488, donkey-anti-rabbit-Cy3, donkey-anti-guinea pig-Cy2, goat-anti-rabbit-Cy3, goat-anti-mouse-Cy2, all polyclonal anti IgG H+L, Dianova. For microscopic analysis, a Leica DMRBE/RD photomicroscope with epifluorescence illumination and a CCD camera (F-View 12) applying Cell software (Olympus) was used. Comparative analysis of basement membrane or M2 macrophage staining were performed using the same exposure time and settings.

### In Situ Zymography

Quenched fluorescein-labeled collagen-IV (Invitrogen, DQ™collagen-IV, bovine skin) were mixed (1∶20) with the EnzChek® Gelatinase/Collagenase Assay Kit (Invitrogen, #E12055) and incubated on unfixed frozen sections at room temperature for 2 h. Sections were fixated (5 min Methanol 4°C, 2 min Acetone −20°C), stained with Hoechst and mounted. The fluorescent signal produced by proteolytic digestion of DQ™collagen-IV was recognized as the combined activity of proteinases capable to cleave collagen-IV (MMP-2, -9, -12, and others). For microscopic analysis, same exposure times and settings were used.

### Gelatin Zymography

Proteins of OTC conditioned media in 2× Sample Buffer (25% 0,5 M Tris/HCl (pH 6,8), 20% Glycerol, 10% SDS, 0,1% Bromphenol Blue solution) were separated in 10% SDS-PAGE gels containing 0,1% gelatin (porcine skin, Fluka). Proteases were renaturated (2,5% Triton X-100) and developed for 24 hours at 37° (50 mmol/L Tris, 5 mmol/L CaCl2, 0.2 mol/L NaCl, and 0,02% Brij). Gel-Staining with Coomassie staining solution (0,5% Coomassie R250, 50% MeOH, 20% acetic acid) was followed by destaining (40% MeOH, 10% acetic acid).

### Quantification

Thickness of dermal equivalents was determined using Photoshop (Adobe, CS3) by measuring the diameter of the collagen gel below the tumor epithelium. Macrophages were quantified manually as macrophage number per visual field. For statistical analysis a two-tailed Mann-Whitney test was performed (GraphPad Prism 4, San Diego) and p<0.05 was considered significant.

## Results

### Macrophages are Viable in OTCs

**Figure 1:**
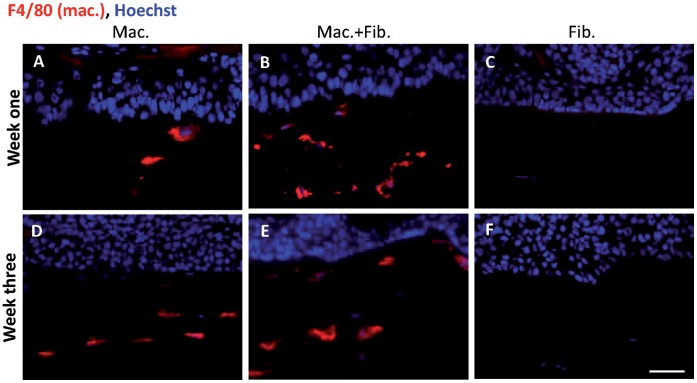
Detection of macrophages in OTCs. Immunofluorescent staining of cryosections against the pan-macrophage marker F4/80 (red) after one and three weeks. F4/80 positive cells are detectable in OTCs in which macrophages had been incorporated alone (week one A, week three D) or together with fibroblasts (week one B, week three E). OTCs in which fibroblasts were integrated were negative for F4/80 (week one C, week three F). Bar 10 µm.

To study the contribution of macrophages in vitro in a more complex, in vivo like situation, we have incorporated murine bone-marrow derived macrophages (BMDM) into organotypic co-cultures of the murine skin SCC cell line PDVA [18] and primary murine dermal fibroblasts. Tumor cells were cultivated air-exposed on top of dermal equivalents consisting of collagen-I containing either BMDM alone or BMDM together with primary dermal fibroblasts. For comparison, dermal equivalents with fibroblasts only were used, as they have been described [10], [11]. To maintain the viability of macrophages, OTCs were stimulated with recombinant murine M-CSF. OTCs were harvested after one and three weeks of culture, cryo-sectioned and analyzed by immunofluorescent stainings for the pan-macrophage marker F4/80. Macrophages could be detected throughout the observation period in OTCs where either macrophages alone (week 1: fig. 1A, week 3: fig. 1D) or together with fibroblasts (week 1: fig. 1B, week 3: fig. 1E) had been incorporated but not in OTCs with fibroblasts only (fig. 1C, F).

### Macrophage Polarization can be Modulated by Exogenous Stimulation

**Figure 2:**
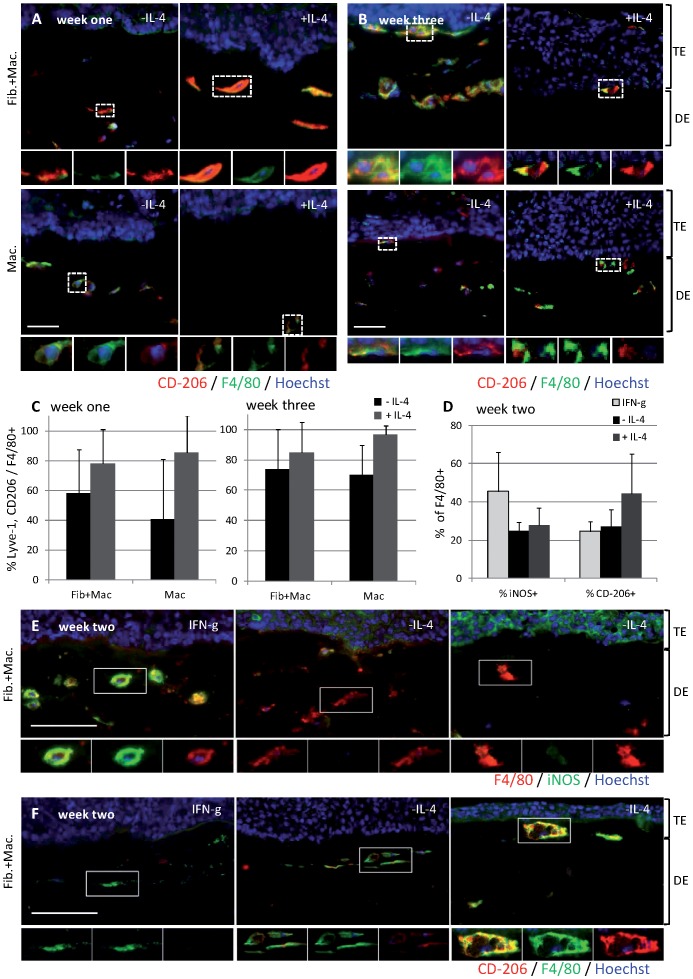
Polarization of macrophages in OTCs. Immunofluorescent staining of cryosections from OTCs against M1 and M2 markers after one and three weeks. M2 polarization of macrophages was detected by staining for the M2 markers CD-206 (A, red). High CD-206 signals were detected in OTCs containing fibroblasts and macrophages or upon treatment with IL-4. CD-206 was detected on macrophages in all 3 week setups (B). Quantification demonstrated that not only signal intensity but also the number of M2 macrophages increased upon stimulation with IL-4 and over time (C). Stimulation of OTCs with IFN-gamma and LPS results in increased numbers of macrophages positive for iNOS, an M1 marker (D), while the number of CD-206+ M2 macrophages decreased (D). Further, the signal intensity of iNOS expression of macrophages in IFN-gamma + LPS stimulated OTCs clearly increased (E), while CD-206 was not detected at all (E). Bar 10 µm.

Macrophages have different effects on tumor growth and progression depending on their activation status. Tumor promoting effects are mediated by M2 polarized macrophages that can be differentiated by IL-4 in vivo and in vitro [22], [23]. We therefore added IL-4 to the growth medium and analyzed macrophage polarization by immunofluorescent staining for the pan macrophage marker F4/80 and the M2 polarization markers Lyve-1 [24] and CD-206 [17]. A small percentage of macrophages in OTCs with fibroblasts and macrophages cultivated in the absence of IL-4 for one week were positive for the M2 markers CD-206 or Lyve-1 (CD-206 fig. 2A, Lyve-1 fig. S1A). In one week old OTCs stimulated with IL-4, a high percentage of macrophages showed high expression of the M2 polarization markers. This was independent of whether macrophages were included into the dermal equivalent together with fibroblasts (fig. 2A CD-206, fig. S1A Lyve-1) or alone (fig. 2 CD-206, fig. S1 Lyve-1). At the same time, no or only weak signals for both Lyve-1 and CD-206 could be detected in OTCs containing macrophages only, cultivated in the absence of IL-4 (fig. 2A). After three weeks of culture, M2 polarized macrophages were detected in all macrophage containing OTCs (fig. 2B, fig. S1B). Neither Lyve-1 nor CD-206 was detected in OTCs containing fibroblasts only (data not shown). Thus, IL-4 stimulation or co-culture with fibroblasts stimulates macrophage M2 polarization after one week. Additionally, macrophages are M2 educated in co-culture with tumor cells after three weeks in absence of fibroblasts and IL-4 stimulation. IL-4 levels were determined in OTCs that were not simulated with IL-4, but no IL-4 could be detected (data not shown). To identify whether macrophages can also be polarized towards an inflammatory M1 phenotype, OTCs were stimulated with IFN-gamma and LPS for one week. Immunofluorescent analysis revealed a strong induction of iNOS, a marker for M1 polarization, in most macrophages (fig. 2E) and an increase in the number of iNOS+ macrophages (fig. 2D), whereas macrophages cultivated in absence of IFN-gamma and LPS with or without IL-4 contained only few macrophages with weak iNOS expression (fig. 2D, E). Expression of the M2 marker CD-206 was not affected by IFN-gamma and LPS treatment but again clearly induced in the IL-4 treated OTCs (fig. 2D, E). While the treatment with IFN-gamma and LPS could clearly induce M1 stimulation, it also negatively affected tumor cell proliferation in absence of macrophages (fig. S3 A) as it has been described before [25], [26]. In contrast IL-4 stimulation had no effect on OTCs that contained fibroblasts but no macrophages. We therefore only analyzed macrophage containing OTCs that were stimulated with IL-4 in detail.

### Macrophages Influence the Tumor Histology of PDVA SCCs

**Figure 3:**
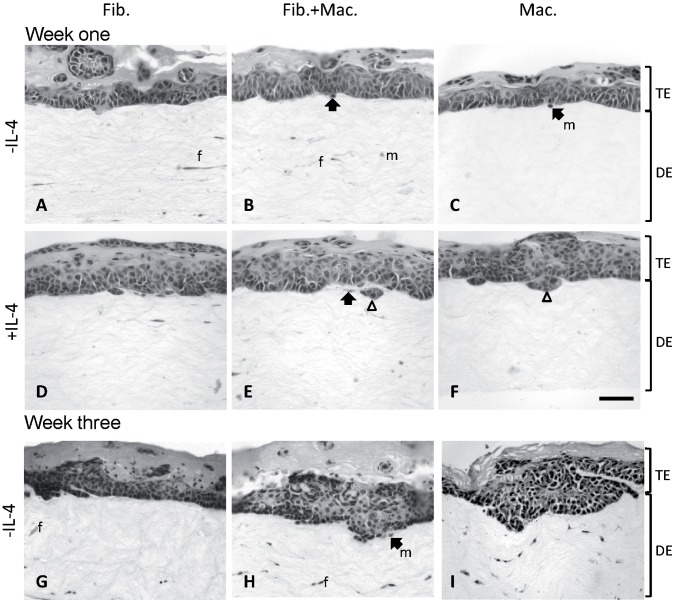
Histology of OTCs containing macrophages. OTCs were fixated with formaldehyde and stained with hemalaun/eosin. PDVA tumor cells form a tumor epithelium (TE) on top of the dermal equivalents (DE). Spindle-shaped fibroblasts (f) and round macrophages (m) were detected within the dermal equivalents. Islands of tumor cells protrude into the dermal equivalents in OTCs containing macrophages in combination with fibroblasts (E) or alone (F) which had been stimulated with IL-4 (bold arrows). After three weeks, infiltrating tumor cells are also observed in OTCs with macrophages in absence of IL-4 (H, I). Bar 10 µm.

To investigate the tumor histology of OTCs in more detail, H&E stainings of OTCs were performed. After one week of culture, PDVA tumor cells had developed a multilayered tumor epithelium on top of the dermal equivalents in all setups (fig. 3 A–F). While OTCs with normal skin keratinocytes develop a stratified epithelium in OTCs [2], PDVA SCC cells formed tumor epithelia which lacked polarization and contained para-keratotic regions. Within the dermal equivalent, spindle-shaped fibroblasts (fig. 3A, marked with f) and rounded macrophages (fig. 3B, marked with m) could be distinguished. Macrophages were often observed in the vicinity of the tumor epithelium (fig. 3 B, C, E, bold arrows). The border between the tumor epithelium and the dermal equivalent was clearly defined and even in all OTCs containing fibroblasts (fig. 3 A, D) and in macrophage containing OTCs cultivated in absence of IL-4 (fig. 3 B, C). In contrast, IL-4 stimulated OTCs containing macrophages showed areas where tumor cells infiltrated the collagen-I gel (fig. 3, E, F) suggesting enhanced invasion of tumor cells. No effect of IL-4 stimulation on fibroblast containing OTCs were observed (fig. 3 D). After three weeks of culture, OTCs containing fibroblasts alone still showed an even tumor-stroma border (fig. 3 G). In contrast, areas of tumor cells infiltrating the collagen-I dermal equivalent were detectable in OTCs containing macrophages and fibroblasts or macrophages alone in absence of IL-4 (fig. 3 H. I).

### M2 Polarized Macrophages Enhance Tumor Cell Invasion

**Figure 4:**
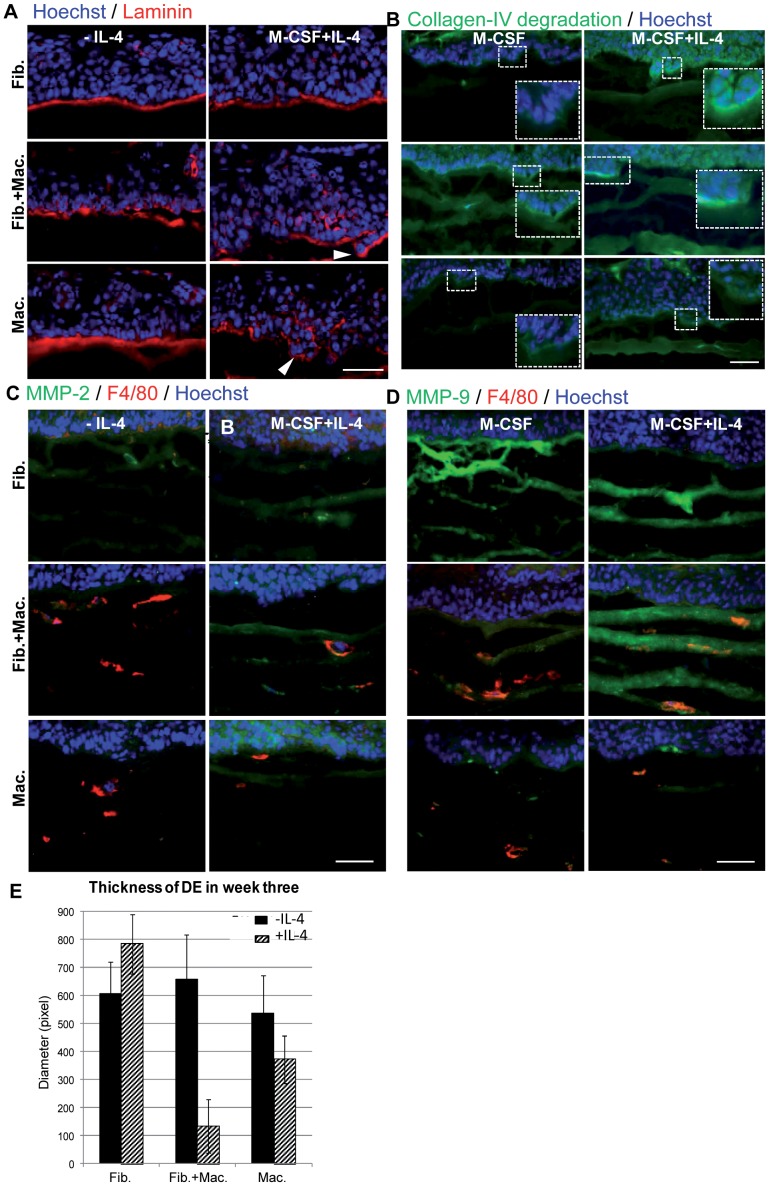
Analysis of tumor cell invasion. A: Immunofluorescent staining for laminin (red), a component of the basement membrane was performed in OTCs cultured for one week. Laminin is disrupted by islands of tumor cells (white arrow heads) in OTCs containing macrophages that were stimulated with IL-4. B: Enzymatic degradation of collagen-IV was analyzed performing *in situ* collagen zymography on OTC cryosections. Collagen-IV can be degraded by MMP-2 and MMP-9. C: Immunofluorescent staining of MMP-2 shows increased signal in macrophage containing OTCs that were stimulated with IL-4 and, to a lesser extent in fibroblast containing OTCs. D: Immunofluorescent staining of MMP-9 reveals increased expression in macrophage containing OTCs stimulated with IL-4 as well as in fibroblast-containing OTCs. E: Thickness of dermal equivalents were quantified. Dermal equivalents are significantly reduced in OTCs containing macrophages or fibroblasts and macrophages stimulated with IL-4. Bar 10 µm.

Tumor cell invasion is characterized by disruption of the basement membrane. We therefore analyzed the structure of the basement membrane in the different OTC settings by immunofluorescent staining against the basement membrane components laminin (fig. 4A) and collagen-IV (fig. S2A) and the basement membrane receptor alpha-6 integrin (fig. S2B). Laminin and collagen-IV stainings were detectable as a layer at the border between the tumor epithelium and the adjacent dermal equivalent in all of the OTCs cultured for one week (fig. 4A, fig. S2A). Alpha-6 integrin staining was most pronounced on the basolateral side but could also be detected suprabasally (fig. S2B). While collagen-IV and laminin stainings were continuous in OTCs containing fibroblasts alone or macrophages alone cultivated in the absence of IL-4, OTCs containing fibroblasts and macrophages that were not stimulated with IL-4 showed an irregular but still continuous staining for laminin and collagen-IV at the tumor-stroma interface. The basement membrane was clearly disrupted in OTCs that contained macrophages and were cultivated in presence of IL-4 (white arrow heads in fig. 4 A, B). Islands of tumor cells that were positive for alpha-6 integrin (fig. S2B) penetrated into the dermal equivalent below the basement membrane zone. Basement membrane components could also be detected within the tumor epithelium. After three weeks of culture, hardly any basement membrane was detected in any of the co-cultures anymore (fig. S2B).

To investigate whether the disruption of the basement membrane observed in OTCs containing M2 polarized macrophages was due to enhanced proteolytic activity, a collagenase assay was performed *in situ* (fig. 4B). While only weak signals of collagen-IV degradation were observed in OTCs containing macrophages (4B, upper row, left) or fibroblasts only (fig. 4B lower row), strong collagenolytic activity was identified in OTCs with macrophages alone or together with fibroblasts that were stimulated with IL-4. Moderately increased collagenolytic activity was observed in OTCs containing fibroblasts together with macrophages that were cultivated in absence of IL-4 (fig. 4B middle panel left) and highest collagen-IV degradation was observed at the interface between tumor cells and the dermal equivalent, especially in those areas where tumor cells protruded into the gel and where disruption of the basement membrane had been observed in consecutive cryosections (fig. 4B top and middle panel right). Immunofluorescent staining of MMP-2 and MMP-9, two gelatinases able to degrade collagen-IV, revealed that enhanced collagenolytic activity was accompanied by enhanced MMP-2 and MMP-9 signal intensity (fig. 4C, D). While MMP-9 was also enhanced in OTCs containing fibroblasts only, MMP-2 was clearly increased in macrophage containing OTCs that were stimulated with IL-4. To further confirm enhanced proteolytic activity in OTCs containing M2 macrophages, we measured the thickness of the entire dermal equivalent below the tumor epithelium in week three in all cultures and found it to be significantly reduced in OTCs containing M2 polarized macrophages (fig. 4E). Taken together, OTCs containing macrophages with either IL-4 stimulation or fibroblast co-cultivation show an irregular staining of the BM components collagen-IV and laminin. This is concomitant with enhanced collagenolytic activity, increased levels of MMP-2 and MMP-9 and reduced thickness of the dermal equivalent.

### Incorporation of Macrophages into OTCs can be Adapted to the Human System

**Figure 5:**
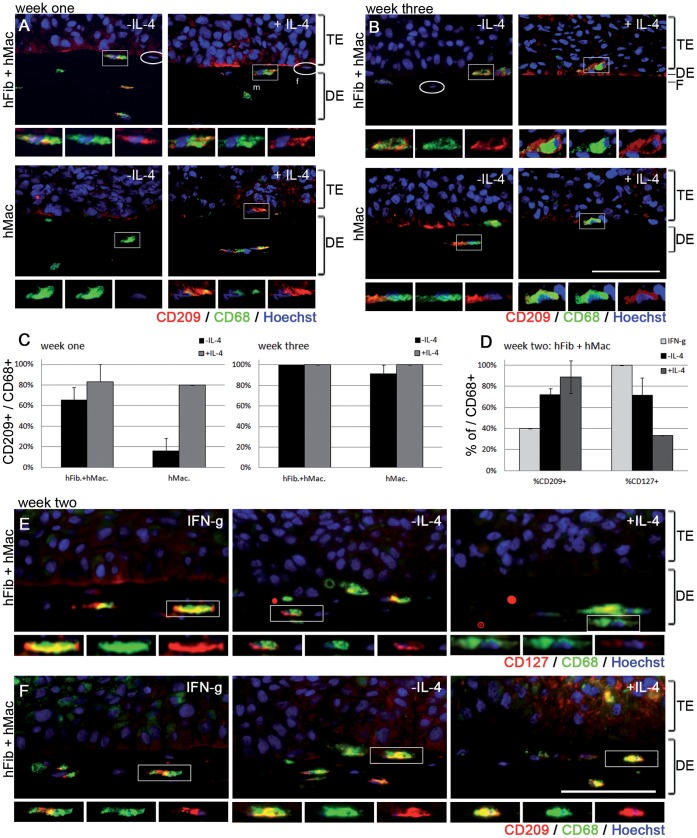
Adaptation of macrophage containing tumor OTCs to a human system. Human PBMC-derived macrophages and human primary dermal fibroblasts were included in OTCs with the human SCC cell line A-5RT3. Immunofluorescent staining of the pan macrophage marker CD-68 (green) and the M2 polarization marker CD-209 (red) in week one (A) and week three (B) shows that macrophages are viable and that in week one, M2 polarization is induced upon co-culture with fibroblasts or upon stimulation with IL-4 (A). Prolonged cultivation for three weeks results in enhanced CD-209 expression in all setups (B). Quantification of CD-209+ macrophages further underlines this dynamics (C). Addition of IFN-gamma and LPS in week two of culture results in increased production of the M1 marker CD-127 (quantification: D, immunofluorescent staining in red: E) whereas unstimulated or IL-4 stimulated OTCs contain less CD-127 macrophages with a lower expression level. Only few CD-209+ macrophages can be detected in OTCs stimulated with IFN-gamma and LPS (F). Bar = 100 µm.

So far, we described the establishment and characterization of a murine tumor OTC model, involving murine bone-marrow derived macrophages, primary murine fibroblasts and a murine SCC cell line. To extend this model system for the investigation in a purely human background, we adapted the model for human cells. As a human skin SCC cell line, we used A-5 RT3 cells and incorporated human primary dermal fibroblasts and human macrophages derived from PBMCs into the dermal equivalent. Immunofluorescent staining against the human pan-macrophage marker CD-68 and the M2 polarization marker CD-209 [27] was performed to confirm the viability and polarization status of macrophages in OTCs. CD-68 positive cells were detected in the dermal equivalents of OTCs containing macrophages only or macrophages together with fibroblasts in week one (fig. 5A) and week three (fig. 5B). Fibroblasts could be clearly distinguished from CD-68 positive macrophages in co-cultures containing both cell types due to the spindle-shaped nuclei of the fibroblasts (oval boxes in fig. 5B, top left panel). Further, CD-68 staining was absent in OTCs containing fibroblasts only (data not shown). As observed in the murine setup, macrophages were again in close vicinity to the tumor epithelium in week one and week three (fig. 5A, B). While macrophages in week one were positive for the M2 polarization marker CD-209 when they were included in OTCs in co-culture with fibroblasts in absence of IL-4 (fig. 5A), OTCs that contained macrophages alone in the dermal equivalent were negative for CD-209 (fig. 5B). Stimulation of OTCs with IL-4 led to induction of the M2 marker CD-209 in macrophages also in absence of fibroblasts (5A).

After three weeks of culture, co-staining of CD-209 with CD-68 was detected in human macrophages in all co-cultures, even in the absence of fibroblasts and IL-4 (fig. 5B), suggesting that like in the murine system, tumor cells were sufficient to induce M2 polarization upon prolonged exposure. Stimulation of OTCs with IL-4 for three weeks did not further increase CD-209 production but enhanced the percentage of M2 macrophages to 100% (fig. 5C). To induce M1 polarization, OTCs were stimulated with IFN-γ and LPS, resulting in strong induction of the M1 marker CD-127 [23], [27] while only few macrophages showed weak CD-127 expression when OTCs were unstimulated or stimulated with IL-4 (fig. 5D). Accordingly, expression and signal intensity of the M2 marker CD-209 was decreased upon IFN-gamma and LPS treatment (fig. 5D, F).

**Figure 6:**
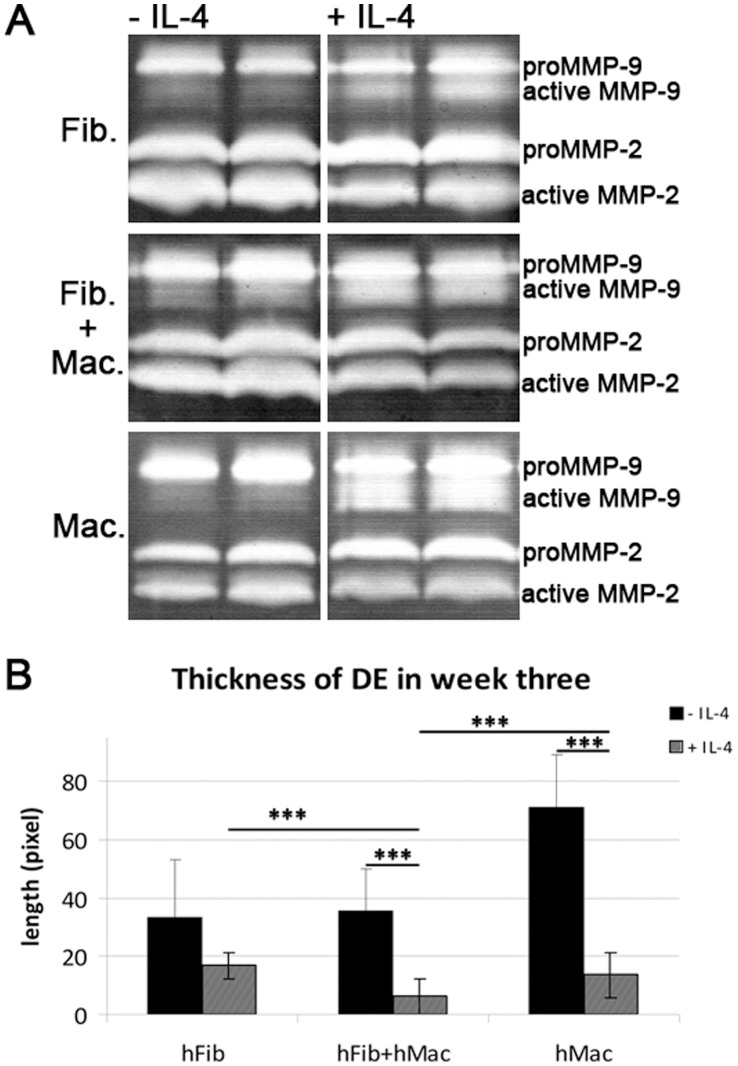
Proteolytic activity in human OTCs. A: Conditioned media from human OTCs were analyzed by gelatin zymography and degradation of co-polimerized gelatin in a SDS PAA gel was investigated. Active MMP-9 is increased in macrophage containing OTCs stimulated with IL-4. B: Thickness of dermal equivalents were quantified. Dermal equivalents are significantly reduced in OTCs containing macrophages or fibroblasts and macrophages stimulated with IL-4.

Immunofluorescent analysis of basement membrane components revealed that human OTCs showed a generally weak basement membrane staining (fig. S4). Yet, fibroblast containing OTCs contained a more pronounced signal for collagen-IV and laminin at the border of the TE to the DE (fig. S4A, B). To identify whether the presence of M2 polarized macrophages in human OTCs also contributes to enhanced proteolytic activity and thus facilitates tumor cell invasion, a gelatin zymography was performed with conditioned medium from OTCs. Activity of proteases in the conditioned medium leads to degradation of co-polimerized gelatin in a SDS PAA gel, which was visualized by coomassie stain. According to their size, pro- and active MMP-2 and MMP-9 were identified and found to be increased in OTCs containing macrophages that were stimulated with IL-4 (fig. 6A). This increased proteolytic activity was associated with a significant reduction in the thickness of dermal equivalents in presence of IL-4, especially when both, macrophages and IL-4 were added (fig. 6B).

Taken together, we successfully established a macrophage-containing tumor OTC in a human background that reproduces features of macrophage activation such as M2 polarization and enhanced proteolytic activity observed in the murine setup.

## Discussion

Tumors resemble organs and develop in a tissue context. Tumor infiltrating cells such as macrophages [15] and fibroblasts [12] as well as ECM components [28] play an important role in tumor progression. In order to study tumor progression in a tissue context in vitro, we have previously described the use of organotypic co-cultures as an in vitro tumorigenicity assay for squamous cell carcinoma [10], [11] where invasion of malignant SCC cell lines was analyzed in an in environment resembling the in vivo situation in presence of fibroblasts. Since macrophages play an important role in tumor progression [15], we now extended this model by including macrophages. The inclusion of macrophages into dermal equivalents has been described before [29], yet their influence on malignant keratinocytes in tumor OTCs had not been characterized. Macrophages were incorporated into the dermal equivalent consisting of collagen-I alone or together with fibroblasts, and skin SCC cells were seeded on top of those dermal equivalent. The system was established in a murine and a human background. Macrophages were viable in the gel after three weeks of culture as evidenced by immunofluorescent staining for F4/80 or CD-68 in the murine or human setup respectively. Since macrophages exert different effects on the tumor microenvironment depending on their activation status [16], we tested whether macrophage polarization in OTCs could be manipulated by addition of exogenous factors. We demonstrated that macrophage polarization in OTCs can be modulated either towards a clear M1 phenotype by stimulation with IFN-γ and LPS, where the majority of macrophages show strong expression of M1 characteristic markers, or towards an M2 phenotype by stimulation with IL-4. In absence of exogenous stimulation, macrophages seemed to initially occur in an intermediate state in the M1/M2 continuum as both, M1 and M2 markers could be detected at low levels.

M2 polarized macrophages were detected in co-cultures stimulated with IL-4 as expected, but also in OTCs containing fibroblasts and macrophages after prolonged culture in absence of IL-4. Thus, while IL-4 clearly induces M2 polarization as described [17], co-cultures of fibroblasts, macrophages and tumor cells also contain factors able to activate macrophages towards an M2 phenotype after prolonged exposure. This interesting aspect was observed in the murine and in the human setup. After three weeks M2 polarized macrophages were also detected in OTCs without fibroblasts that were not treated with IL-4 in both setups, implying an activation of macrophages that occurs over time by tumor-cell derived factors and thus might resemble the macrophage activation dynamics observed in the more complex tumor stroma in vivo. However, spontaneous macrophage polarization in these organotypic co-cultures must be mediated by factors other than IL-4 since no IL-4 could be detected by ELISA in unstimulated cultures. By now, several factors have been identified that mediate alternative macrophage polarization such as Activin-A [30], IL-21 [31], or a combination of CCL-2 and IL-6 [32]. 2009). We have indication that IL-6 and CCL-2 levels are increased in our OTC tumor model. A detailed characterization of spontaneous macrophage polarization in this model is subject of current studies in our lab.

Histological analysis of murine OTCs showed the development of an epithelium of tumor cells on top of the dermal equivalent. However, the epithelium lacked the polarization of a stratified epithelium that is observed in skin OTCs of healthy keratinocytes [2]. Macrophages were often observed in the direct vicinity of the tumor-stroma border. Interestingly, Pollard and co-workers have described that macrophages are often found at the invasive front in breast cancer models, where they facilitate tumor cell migration and invasion by the secretion of soluble factors [33]. Thus, it can be hypothesized that macrophages are recruited to close vicinity of the tumor epithelium by tumor-cell derived factors in OTCs where they facilitate tumor cell invasion by secretion of soluble factors and proteases at the site of invasion comparable to the situation in vivo.

Regions of tumor cells protruding into the gel were observed in week one in murine OTCs containing M2 polarized macrophages, pointing to an enhanced tumor cell invasion. It has previously been described that OTCs with malignant keratinocytes provide a tool to study basement membrane formation and disruption [34]. Analysis of the basement membrane components revealed two interesting findings: i) localization of basement membrane components within the tumor epithelium that underlines the disturbed epithelial polarization in our OTC system as it is commonly observed in malignancies [12] and is associated with increased tumor stiffness which in turn corresponds to enhanced tumor malignancy [35], [36]. ii) We clearly observed a disruption of the basement membrane in several OTC setups. Disruption of the basement membrane by islands of tumor cells protruding into the gel was observed in murine OTCs with macrophages cultured in presence of IL-4, while corresponding OTCs in the human setup showed a generally reduced basement membrane staining. Additionally, the diameter of collagen-I dermal equivalents were reduced after three weeks in OTCs containing macrophages that had been stimulated with IL-4. IL-4 has also been described to act on fibroblasts by increasing extracellular matrix production and plays a causal role in fibrosis [37]. However, the enhanced invasiveness observed in macrophage containing OTCs stimulated with IL-4 was not detected in OTCs containing IL-4 stimulated fibroblasts alone. Thus, we conclude that the observed effects were indeed due to activation of macrophages by IL-4 and not due to effects of IL-4 on tumor cells or fibroblasts. Histological analysis of murine OTCs in week three had shown that macrophage containing cultures cultivated in absence of IL-4 showed enhanced areas of tumor cells protruding into the dermal equivalent. This together with our observation that macrophages underwent M2 polarization in week three even in the absence of IL-4 further supports our finding that the presence of M2 polarized macrophages enhance tumor cell invasion. This is mediated by enhanced proteolytic activity as evidenced by in situ collagenase assay. Specifically, expression and activation of MMP-2 and MMP-9 was upregulated in macrophage containing OTCs stimulated with IL-4, as evidenced by immunofluorescent staining and gelatin zymography. We hypothesize that enhanced levels of both, MMP-2 and MMP-9 accelerate disruption of the basement membrane and degradation of the dermal equivalent, both events that resemble tumor invasion in vivo. Yet, MMPs and probably also other proteases are also present in absence of M2 polarized macrophages, allowing for invasion of tumor cells in these setups as well, yet at a diminished rate. Tumor cell motility seemed to rely on collective cell migration [38] rather on single cell movement, as the tumor cells remain their cell-cell-connections. This is in line with our previous analysis of skin SCC cell behavior in a 2D scratch assay in vitro [39]. Thus, when the basement membrane is mostly intact, as was the case in murine macrophage containing OTCs in week one, tumor cell invasion into the gel only occurs at sites of local basement membrane disruption. In contrast, at later time points or in human OTCs where less basement membrane components were detectable, tumor cells degrade the dermal equivalent and move collectively into the dermal equivalent.

Taken together, macrophage containing tumor-OTCs offer a powerful model to study the influence of tumor stroma interactions on tumor cell invasion in detail under in vivo like conditions. Demonstration that this model can be adapted for a human system further extends its applicability to study the activation of human macrophages in detail. Descriptions of organotypic co-cultures containing macrophages include description of dermal equivalents modeling healthy skin [29] and the culture of tissue explants containing macrophages for the testis [40], neuroretina [41], hippocampal slices [42], and tumor [4]. The organotypic tumor model described here, however, has the great advantage over the described models to be independent of the inter-individual differences that can be a problem in explants cultures and to allow incorporation of macrophages of different origin. While the murine OTC model might be used to dissect the influence of individual cytokines on macrophage activation by using macrophages from transgenic mice, the human model allows incorporation of macrophages derived from patient tumor material or to compare macrophage activation within a human system. We therefore have developed a powerful tool to study the influence of cellular interactions between tumor and stromal cells, including tumor-associated macrophages, in a complex in vivo like environment.

## Supporting Information

Figure S1 **Immunofluorescent analysis of Lyve-1, another M2 polarization marker in macrophage containing murine OTCs.** A: High Lyve-1 signals were detected in OTCs containing fibroblasts and macrophages or upon treatment with IL-4. CD-206 was detected on macrophages in all 3 week setups (B).
(TIF)

Figure S2 **Analysis of the basement membrane in murine OTCs.** A: Immunofluorescent staining for collagen-IV (red), a component of the basement membrane was performed in OTCs cultured for one week. Collagen-IV was disrupted by islands of tumor cells (white arrow heads) in OTCs containing macrophages that were stimulated with IL-4. B: Alpha-6 integrin was localized around tumor cells in the basal as well as in subrabasal layer (arrow) and showed the strongest signal at the basolateral side of the tumor epithelium (arrow head). Alpha-6 integrin+ tumor cells could be detected that protruded into the dermal equivalent. C: Immunofluorescent staining of collagen-IV (red) in cryosections shows that collagen-IV can be detected within the tumor epithelium rather than at the tumor-stroma border after three weeks of culture. D: Immunfluorescent staining of laminin (red) reveals that only little laminin is detectable after three weeks of culture.
(TIF)

Figure S3 **Tumor cell proliferation in murine and human OTCs.** A: Immunofluorescent analysis of murine OTCs. Proliferating tumor cells (Ki-67+, red) were detected throughout the tumor epithelium. The number of proliferating tumor cells increased in macrophage containing OTCs stimulated with IL-4 but decreased upon IFN-gamma and LPS treatment, independent of the cellular components of the dermal equivalent. B: Immunofluorescent analysis of human OTCs revealed again reduced tumor cell proliferation upon IFN-gamma and LPS treatment.
(TIF)

Figure S4 **Analysis of the basement membrane in one week old human OTCs.** Immunofluorescent analysis of collagen-IV (A) and laminin (B) showed that human OTCs did not develop a continuous basement membrane. Yet, in presence of fibroblasts only, collagen-IV expression was stronger than in macrophage containing OTCs and laminin localization at the basement membrane was increased. Alpha-6 integrin (C) could again be detected suprabasally as well as at the basolateral side. Bar = 100 µm.
(TIF)
