# Supplementary figures and images for: Integrating Macrophages into Organotypic Co-Cultures: A 3D In Vitro Model to Study Tumor-Associated Macrophages
Source: PLoS One. 2012 Jul 6;7(7):e40058. doi: 10.1371/journal.pone.0040058 (PMC3391227; doi:10.1371/journal.pone.0040058)

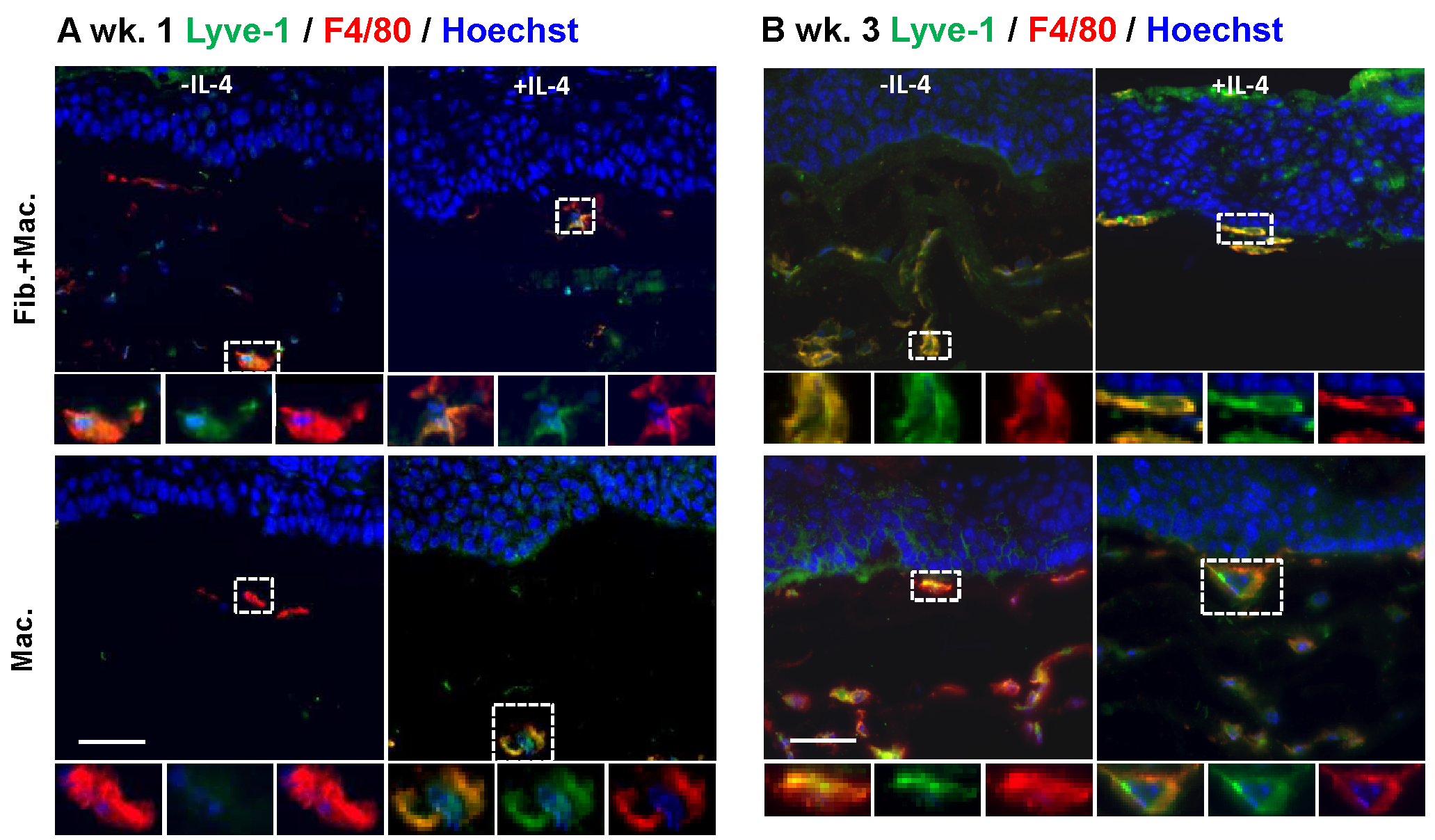

Supplement: Figure S1 — Immunofluorescent analysis of Lyve-1, another M2 polarization marker in macrophage containing murine OTCs. A: High Lyve-1 signals were detected in OTCs containing fibroblasts and macrophages or upon treatment with IL-4. CD-206 was detected on macrophages in all 3 week setups (B). (TIF) [file pone.0040058.s001.tif]

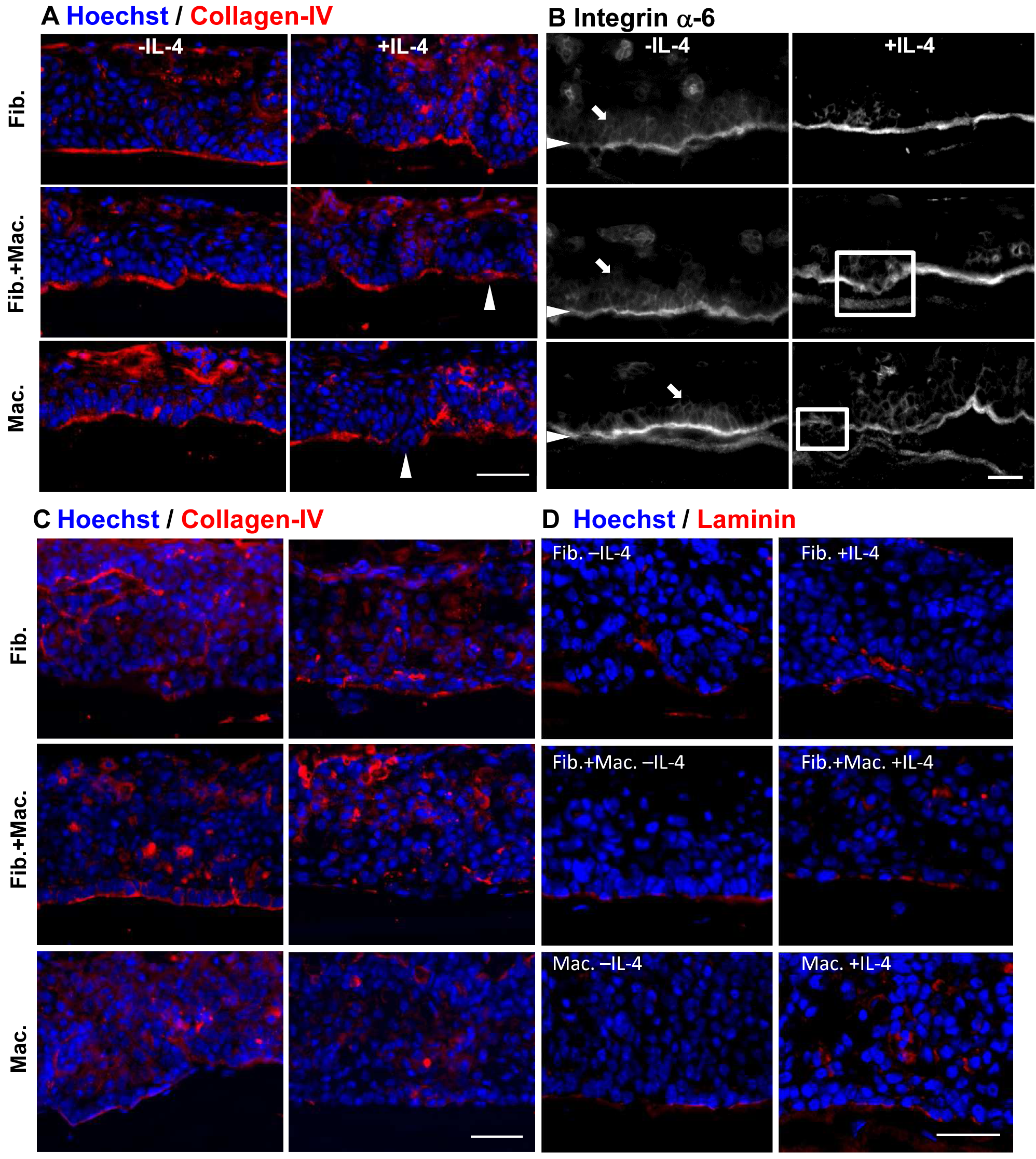

Supplement: Figure S2 — Analysis of the basement membrane in murine OTCs. A: Immunofluorescent staining for collagen-IV (red), a component of the basement membrane was performed in OTCs cultured for one week. Collagen-IV was disrupted by islands of tumor cells (white arrow heads) in OTCs containing macrophages that were stimulated with IL-4. B: Alpha-6 integrin was localized around tumor cells in the basal as well as in subrabasal layer (arrow) and showed the strongest signal at the basolateral side of the tumor epithelium (arrow head). Alpha-6 integrin+ tumor cells could be detected that protruded into the dermal equivalent. C: Immunofluorescent staining of collagen-IV (red) in cryosections shows that collagen-IV can be detected within the tumor epithelium rather than at the tumor-stroma border after three weeks of culture. D: Immunfluorescent staining of laminin (red) reveals that only little laminin is detectable after three weeks of culture. (TIF) [file pone.0040058.s002.tif]

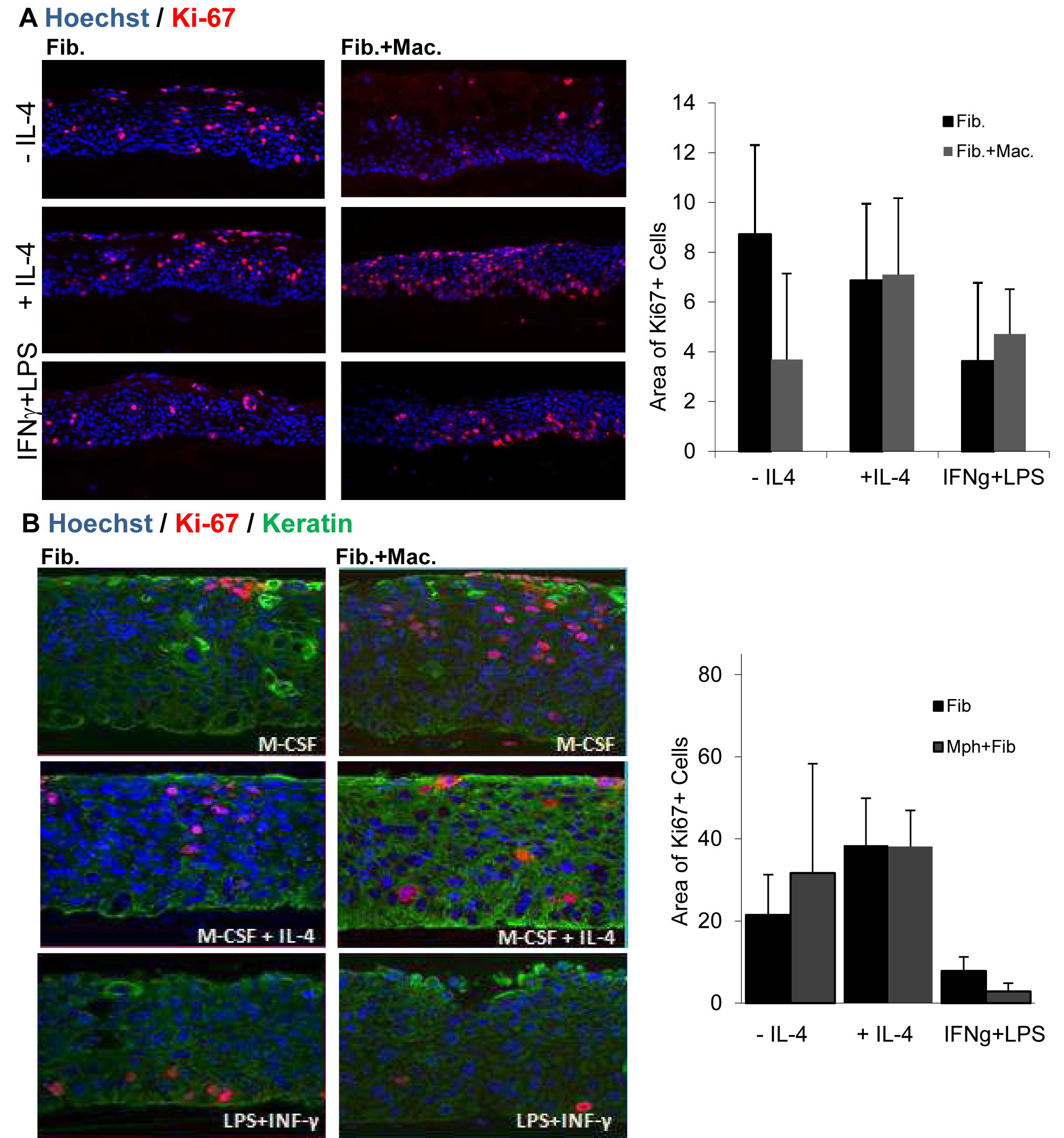

Supplement: Figure S3 — Tumor cell proliferation in murine and human OTCs. A: Immunofluorescent analysis of murine OTCs. Proliferating tumor cells (Ki-67+, red) were detected throughout the tumor epithelium. The number of proliferating tumor cells increased in macrophage containing OTCs stimulated with IL-4 but decreased upon IFN-gamma and LPS treatment, independent of the cellular components of the dermal equivalent. B: Immunofluorescent analysis of human OTCs revealed again reduced tumor cell proliferation upon IFN-gamma and LPS treatment. (TIF) [file pone.0040058.s003.tif]

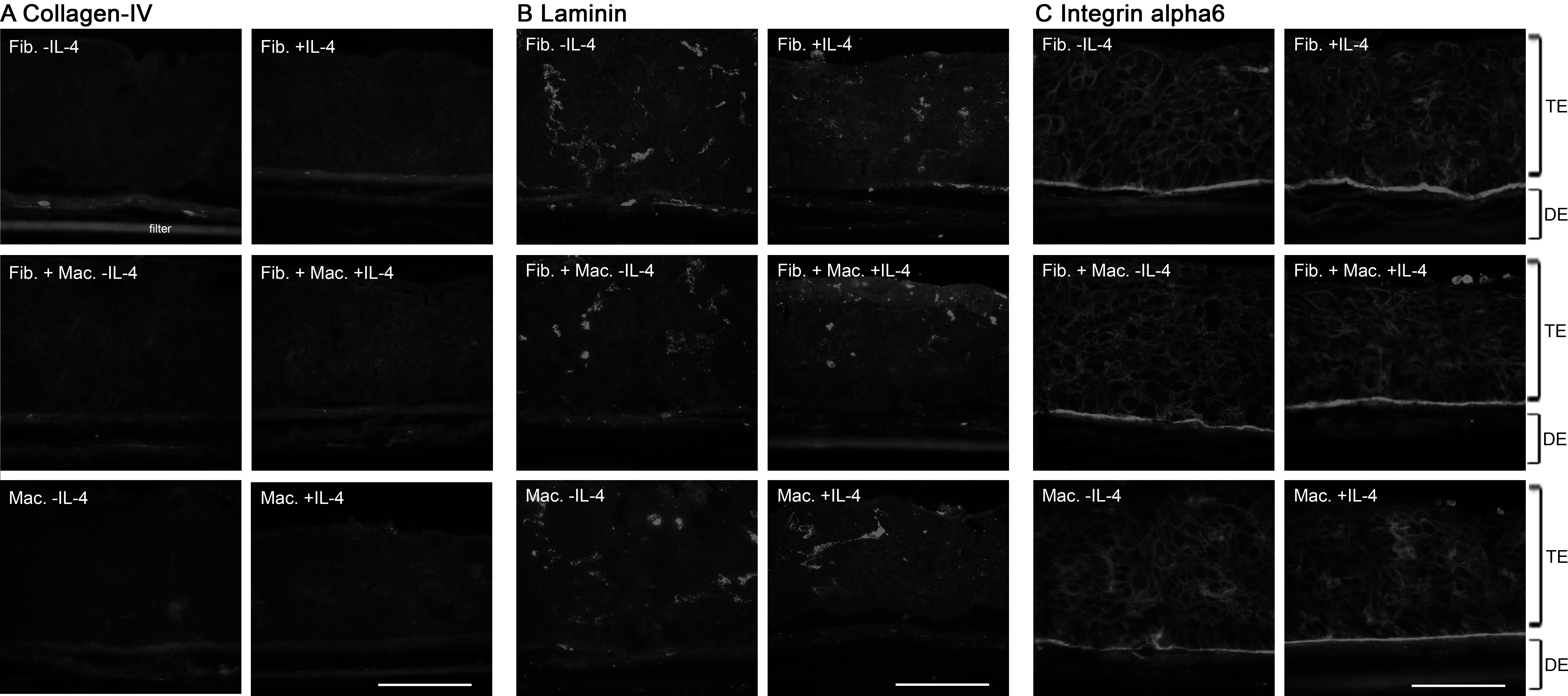

Supplement: Figure S4 — Analysis of the basement membrane in one week old human OTCs. Immunofluorescent analysis of collagen-IV (A) and laminin (B) showed that human OTCs did not develop a continuous basement membrane. Yet, in presence of fibroblasts only, collagen-IV expression was stronger than in macrophage containing OTCs and laminin localization at the basement membrane was increased. Alpha-6 integrin (C) could again be detected suprabasally as well as at the basolateral side. Bar = 100 µm. (TIF) [file pone.0040058.s004.tif]
